# Proteolysis of *Xenopus* Cip-type CDK inhibitor, p16^Xic2^, is regulated by PCNA binding and CDK2 phosphorylation

**DOI:** 10.1186/1747-1028-8-5

**Published:** 2013-04-22

**Authors:** Xi-Ning Zhu, Dong Hyun Kim, Horng-Ru Lin, Varija N Budhavarapu, Herbert B Rosenbaum, Paul R Mueller, P Renee Yew

**Affiliations:** 1 Department of Molecular Medicine, Institute of Biotechnology, The University of Texas Health Science Center at San Antonio, San Antonio, TX 78229, USA; 2 Department of Biology, The University of Texas at San Antonio, San Antonio, TX, 78249, USA; 3 Current address: DiaCarta Inc., P.O. Box 360772, Milpitas, CA, 95036, USA; 4 Current address: Department of Infectious Diseases, Novartis Institute for Biomedical Research, 4560 Horton Street MS 4.3, Emeryville, CA, 94608-2916, USA; 5 Current address: MD Anderson Cancer, Department of Biochemistry and Molecular Biology, University of Texas, Houston, TX, 77030, USA; 6 Department of Molecular Medicine, Institute of Biotechnology, Mail Code 8257, South Texas Research Facility, The University of Texas Health Science Center at San Antonio, 7703 Floyd Curl Drive, San Antonio, TX 78229-3900, USA

**Keywords:** Xic2, *Xenopus*, PCNA, Phosphorylation, Proteolysis, CDK inhibitor

## Abstract

**Background:**

Cell division is positively regulated by cyclin-dependent kinases (CDKs) partnered with cyclins and negatively regulated by CDK inhibitors. In the frog, *Xenopus laevis*, three types of CDK inhibitors have been described: p27^Xic1^ (Xic1) which shares sequence homology with both p21^Cip1^ and p27^Kip1^ from mammals, p16^Xic2^ (Xic2) which shares sequence homology with p21^Cip1^, and p17^Xic3^ (Xic3) which shares sequence homology with p27^Kip1^. While past studies have demonstrated that during DNA polymerase switching, Xic1 is targeted for protein turnover dependent upon DNA, Proliferating Cell Nuclear Antigen (PCNA), and the ubiquitin ligase CRL4^Cdt2^, little is known about the processes that regulate Xic2 or Xic3.

**Methods:**

We used the *Xenopus* interphase egg extract as a model system to examine the regulation of Xic2 by proteolysis and phosphorylation.

**Results:**

Our studies indicated that following primer synthesis during the initiation of DNA replication, Xic2 is targeted for DNA- and PCNA-dependent ubiquitin-mediated proteolysis and that Cdt2 can promote Xic2 turnover. Additionally, during interphase, Xic2 is phosphorylated by CDK2 at Ser-98 and Ser-131 in a DNA-independent manner, inhibiting Xic2 turnover. In the presence of double-stranded DNA ends, Xic2 is also phosphorylated at Ser-78 and Ser-81 by a caffeine-sensitive kinase, but this phosphorylation does not alter Xic2 turnover. Conversely, in the presence or absence of DNA, Xic3 was stable in the *Xenopus* interphase egg extract and did not exhibit a shift indicative of phosphorylation.

**Conclusions:**

During interphase, Xic2 is targeted for DNA- and PCNA-dependent proteolysis that is negatively regulated by CDK2 phosphorylation. During a response to DNA damage, Xic2 may be alternatively regulated by phosphorylation by a caffeine-sensitive kinase. Our studies suggest that the three types of *Xenopus* CDK inhibitors, Xic1, Xic2, and Xic3 appear to be uniquely regulated which may reflect their specialized roles during cell division or early development in the frog.

## Background

The vertebrate cell cycle is positively regulated by cyclin-dependent kinases (CDKs) and negatively regulated by CDK inhibitors [1]. Vertebrate CDK inhibitors of the Cip/Kip-type bind to and negatively regulate CDK2-cyclins E/A and the onset of DNA replication [2]. Cip-type CDK inhibitors also bind to and negatively regulate the replication protein, Proliferating Cell Nuclear Antigen (PCNA) [3]. Studies have indicated that mammalian Cip/Kip-type CDK inhibitors are frequently targeted for ubiquitin-mediated protein turnover during the G1 to S phase transition resulting in the activation of CDK2-cyclins and the progression into S phase [4-8].

In the frog, *Xenopus laevis*, three Cip/Kip-type CDK inhibitors have been identified that share sequence homology with mammalian p21^Cip1^ (p21), p27^Kip1^ (p27), and p57^Kip2^ (p57) [9-11]. p27^Xic1^/p28^Kix1^ (Xic1/Kix1) shares homology with all three of the mammalian Cip/Kip-type CDK inhibitors while p16^Xic2^ (Xic2) is more closely related to p21 and p17^Xic3^ (Xic3) is more closely related to p27 [9-11]. Developmental studies suggest that Xic1 is the only CDK inhibitor that is expressed in the early embryo and studies indicate that Xic1 is required for both the differentiation of nerve and muscles cells [11-18]. The expression of both Xic2 and Xic3 appears to be more tissue-specific in nature with Xic2 found in somites, the tail bud, lens, and the cement gland while Xic3 is expressed primarily in the central nervous system [11]. While overexpression of Xic2 and Xic3 in the developing embryo results in an arrest in cell division due to an inhibition of CDK2 activity, little is known about the possible regulatory pathways that may control the activities of Xic2 or Xic3 [11].

Using the *Xenopus* interphase egg extract as a model biochemical system to study DNA replication initiation and CDK inhibitor regulation, studies have demonstrated that Xic1 is targeted for ubiquitination by the ubiquitin ligase, CRL4^Cdt2^, in a DNA- and PCNA-dependent manner during DNA polymerase switching resulting in its degradation [19-21]. In an effort to understand the possible molecular mechanisms that may regulate Xic2 and Xic3, we have taken a similar approach and used the *Xenopus* interphase egg extract as a biochemical model system to study Xic2 and Xic3. Our results suggest that while Xic3 appears to be stable in the *Xenopus* extract, Xic2 is targeted for ubiquitination and phosphorylation in the extract in a manner that is dependent upon specific DNA templates.

## Results

### *Xenopus* Cip/Kip-type CDK inhibitors are differentially modified in the interphase egg extract

**Figure 1:**
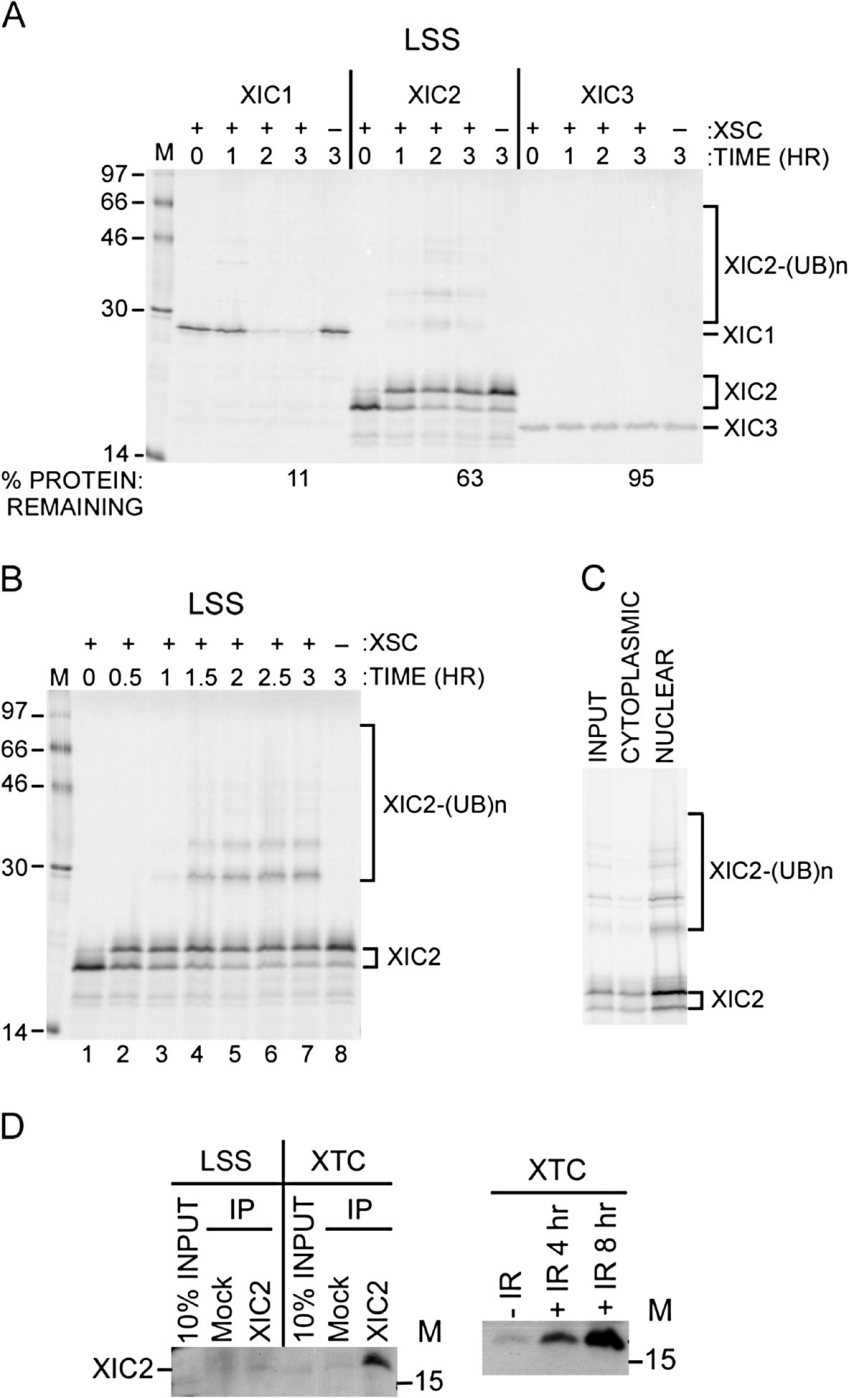
**Xic2 is ubiquitinated and degraded in a DNA dependent manner. A**. Degradation assay. ^35^S-methionine labeled Xic1, Xic2, and Xic3 were incubated in *Xenopus *interphase egg extract (Low Speed Supernatant, LSS) in the absence (-) or presence (+) of *Xenopus *sperm chromatin (XSC) for 0 to 3 hours as indicated. The mean percentage of remaining protein from two independent experiments is shown (% protein remaining) where the zero hour time point was normalized to 100%. Xic1, Xic2, and Xic3 protein bands are marked on the right including ubiquitinated [(UB)n] forms of Xic2. **B**. Xic2 ubiquitination assay. ^35^S-methionine labeled Xic2 was incubated in LSS with methyl ubiquitin (3 mg/ml) to stabilize the ubiquitinated species in the absence (-) or presence (+) of XSC for 0 to 3 hours as indicated. Xic2 protein bands are marked on the right including ubiquitinated [(UB)n] forms. **C**. Nuclei spin down assay. Nuclei spin down assay was employed to separate cytosolic (CYT) and nuclear fractions (NUC) after incubation of [^35^S]-methionine labeled Xic2 with LSS containing XSC. The input (INPUT) represents 1/15th of the sample before centrifugation and the cytosolic (CYT) represents 1/15^th^ of the cytosolic fraction after centrifugation. Xic2-(UB) n denotes ubiquitinated Xic2. **D**. Xic2 immunoblot. Left panel: *Xenopus *LSS or XTC cell extracts were immunoprecipitated (IP) using anti-Xic2 (XIC2) or normal rabbit serum (Mock) antibody and then immunoblotted with anti-Xic2 antibody. Ten percent of the immunoprecipitation reaction was loaded directly (10% INPUT). Right panel: XTC cells were treated with gamma irradiation (IR, 10 Gy) and harvested 4 or 8 hrs following treatment. Lysates were then examined by immunoblotting with anti-Xic2 antibody. For all figures, the molecular weight marker (M) is shown in kilodaltons.

To study the regulation of the *Xenopus* CDK inhibitor, Xic1, we have previously used the biochemically tractable egg extract as a model system [19]. In these studies, we have dissected the molecular mechanism of Xic1 turnover and have found that Xic1 is degraded in the egg extract during DNA polymerase switching in a DNA-, PCNA-, and CRL4^Cdt2^-dependent manner [19-23]. CRL4^Cdt2^ is a member of the Cullin-RING-type ubiquitin ligases which includes CRL1^Skp2^, previously shown to ubiquitinate Xic1 in vitro [24]. Using the interphase egg extract, we found that Xic3 was completely stable in the egg extract, Xic2 was partially degraded and partially modified in a manner resembling ubiquitination and/or phosphorylation (Figure 1A), and Xic1 was readily degraded as shown in previous studies [21]. The Xic2 modification resembling ubiquitination appeared to be DNA-dependent while the putative phosphorylation of Xic2 (band migrating at ~22 kDa) was not dependent upon the presence of DNA (Figure 1A). To further examine the modified species of Xic2, we added methyl ubiquitin to stabilize monoubiquitination and prevent polyubiquitination [25] and found that the higher molecular weight forms of Xic2 were stabilized indicating that they represent monoubiquitinated Xic2 species (Figure 1B). We also noticed that while the unmodified form of Xic2 decreased as the ubiquitinated forms of Xic2 increased, the modified form of Xic2 which may represent phosphorylated Xic2 remained stable (Figure 1B). Cellular localization studies indicated that both the ubiquitinated forms and the putative phosphorylated form of Xic2 were localized predominantly to the nucleus (Figure 1C) [23]. These studies suggest that the unmodified form of Xic2 can be degraded by a DNA and ubiquitin-dependent pathway in the interphase egg extract while the putative phosphorylated form of Xic1 may be resistant to ubiquitination and degradation.

To examine Xic2 in a more physiological manner, we generated an antibody to Xic2 and immunoblotted the Xic2 protein in the *Xenopus* interphase egg extract and in *Xenopus* Tissue Culture (XTC) cells. We found that Xic2 was present at very low levels in the interphase egg extract following immunoprecipitation and immunoblotting (Figure 1D, left panel), while in XTC cells, Xic2 was readily detectable as a single protein band (Figure 1D, left panel). Moreover, we found that following ionizing irradiation (IR) of XTC cells, the expression of Xic2 was greatly increased and was easily detectable by direct immunoblotting (Figure 1D, right panel). This result suggests that Xic2 is not highly expressed in the early embryo, but becomes more highly expressed in somatic cells. Additionally, this result suggests that like mammalian p21 [26], Xic2 is highly induced following exposure to IR.

### Xic2 is uniquely phosphorylated during the cell cycle and in response to single-stranded DNA

**Figure 2:**
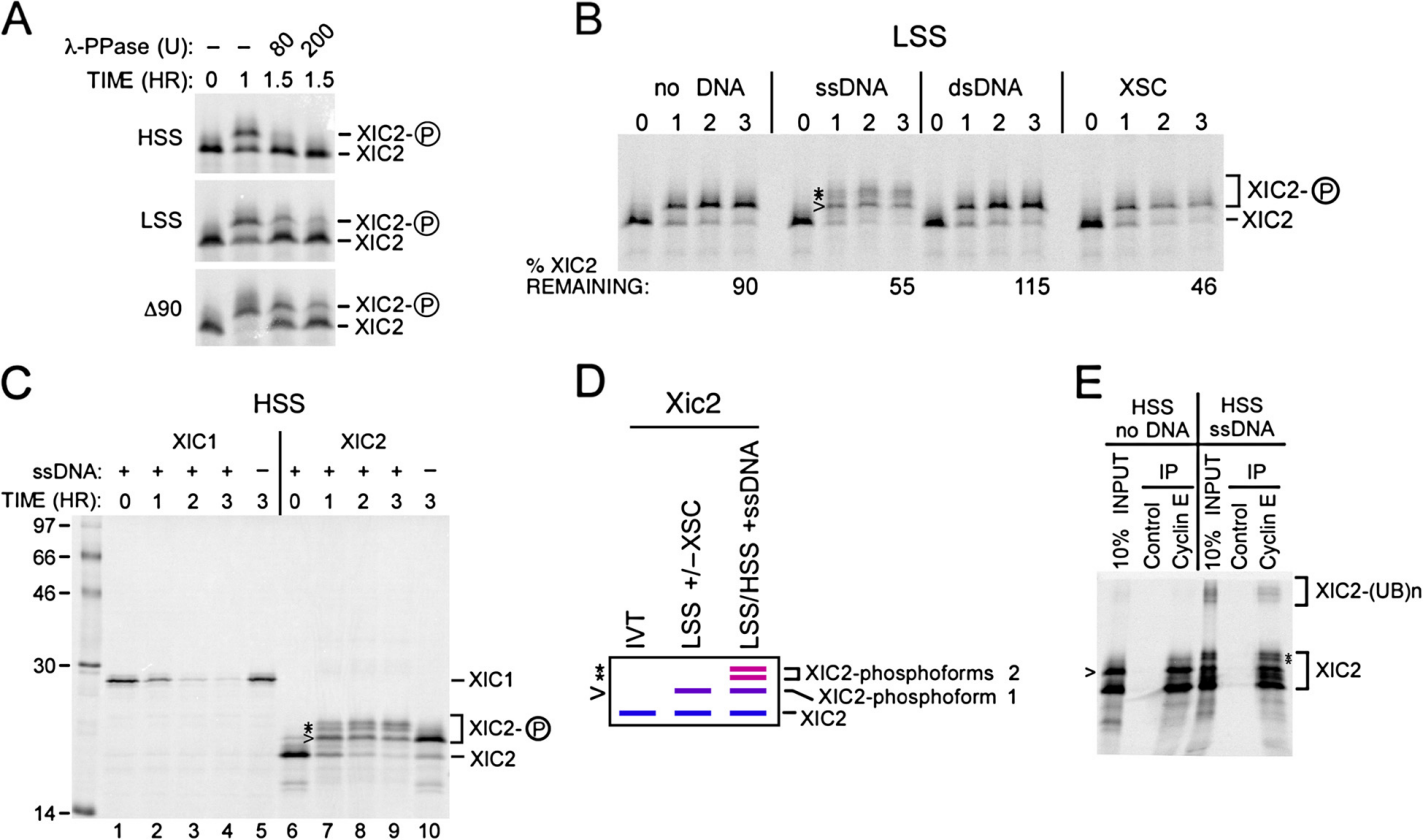
**Xic2 is differentially phosphorylated in the absence and presence of single-stranded DNA. A**. Xic2 phosphorylation shift assay. ^35^S-methionine labeled Xic2 was incubated in interphase egg extract (LSS or HSS) or mitotic extract (Δ90) as indicated in the absence (-) or presence of 80 or 200 units (U) of lambda phosphatase (λ-PPase). **B**. Xic2 phosphorylation shift and degradation assay. ^35^S-methionine labeled Xic2 was incubated in LSS with buffer (no DNA), single-stranded ΦX174 DNA (ssDNA, 10 ng/ul), pCS2+ plasmid DNA (dsDNA, 10 ng/ul), or XSC (10 ng/ul) at 23°C. Samples were analyzed by SDS-PAGE at 0-3 hrs. The mean percentage of remaining Xic2 from two independent experiments is shown (% Xic2 remaining) where the zero hour time point was normalized to 100%. **C**. Xic2 degradation assay. ^35^S-methionine labeled Xic1 or Xic2 was incubated in HSS with (+) or without (-) single-stranded DNA (ssDNA) for 0–3 hrs as indicated. Molecular weight markers are shown in kilodaltons. **D**. Schematic representation of ^35^S-methionine labeled Xic2 phosphoforms in the absence of extract or DNA (IVT) (left lane), in the presence of LSS with or without XSC (middle lane), or in the presence of LSS or HSS with ssDNA (right lane). Unphosphorylated Xic2 is marked by the blue line (XIC2), Xic2 phosphoform 1 is marked by the purple line and the caret (>), and Xic2 phosphoforms 2 are marked by the pink lines and the asterisks (*). **E**. Xic2 co-immunoprecipitation with cyclin E. ^35^S-methionine labeled Xic2 was incubated in HSS without (no DNA) or with (ssDNA) as indicated. Xic2 was co-immunoprecipitated (IP) with anti-cyclin E or control antibody. 10% of the input reaction is shown (10% INPUT). In all figures, “XIC2-P” or the caret (<) and asterisks (*) indicate the phosphoforms of Xic2 and ubiquitinated Xic2 protein bands are indicated as “XIC2-(UB)n”.

To characterize the nature of the DNA-independent modified Xic2 species, we examined Xic2 in the membrane-free interphase high speed supernatant (HSS), the membrane-containing interphase LSS, and a stable mitotic extract (Δ90 extract) generated by supplementing interphase extract with non-degradable cyclin B [19,21]. In the absence of DNA, we observed a putative phosphoform of Xic2 in both the HSS and LSS that was reversed by phosphatase treatment (Figure 2A). Moreover, multiple Xic2 phosphoforms observed to be diminished by phosphatase were present in the mitotic extract suggesting potentially multiple sites of Xic2 phosphorylation during mitosis (Figure 2A). Similar studies resulted in no detectable shift of the Xic3 protein band in either interphase or mitotic egg extracts (data not shown) while previous studies have demonstrated that Xic1 is phosphorylated in the mitotic extract [9,19]. To further examine the phosphorylation of Xic2 and the DNA-dependent turnover of Xic2, we examined Xic2 in different extract systems with alternative DNA templates. In the membrane-containing LSS, Xic2 was stable in the absence of DNA and in the presence of super-coiled plasmid DNA, but was roughly 50% degraded in the presence of single-stranded DNA and sperm chromatin (Figure 2B). As noted above, Xic2 exhibited a single DNA-independent phosphoform in the interphase extract which we term “phosphoform 1” (Figure 2B and D). Curiously, in addition to phosphoform 1, Xic2 exhibited at least two additional shifts we term “phosphoforms 2” in both the LSS and HSS only in the presence of single-stranded DNA (Figure 2B, C, and D). To determine if any of the modified forms of Xic2 altered Xic2’s ability to associate with cyclin E in the egg extract, we performed an immunoprecipitation reaction using anti-cyclin E antibody and found that all the modified forms of Xic2 could readily associate with cyclin E (Figure 2E).

### Xic2 turnover is dependent upon PCNA and PCNA binding

**Figure 3:**
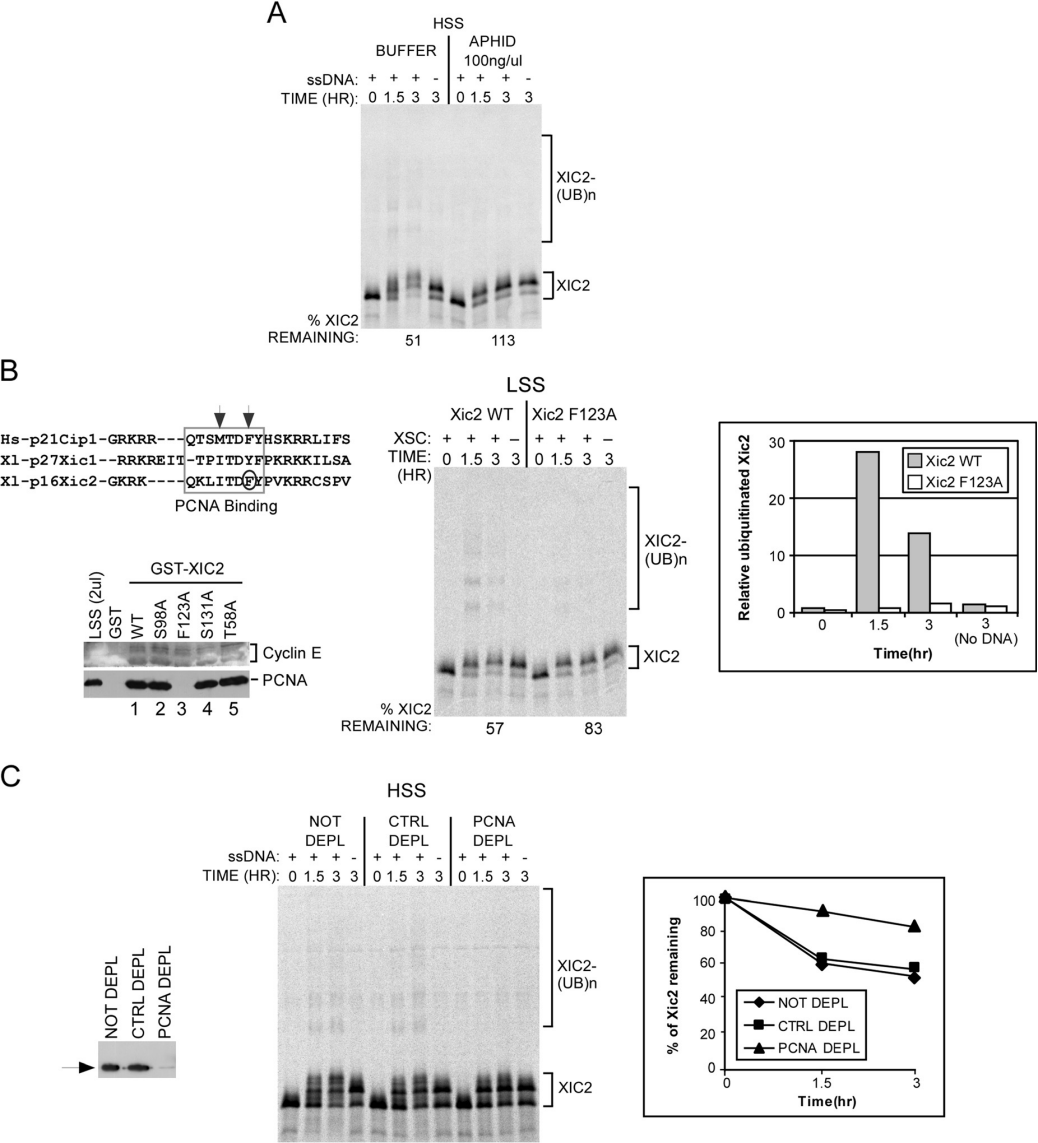
**Xic2 is degraded in a PCNA-dependent manner. A**. Xic2 degradation assay. ^35^S-labeled Xic2 was incubated in HSS with (+) or without (-) ssDNA (ΦX174) with buffer (methanol) or aphidicolin (100 ng/ul). The mean percentage of remaining Xic2 from two independent experiments is shown (% Xic2 remaining) where the zero hour time point was normalized to 100%. **B**. Left top panel: Sequence alignment of the PCNA binding domain in human p21^Cip1^, *Xenopus *p27^Xic1^, and *Xenopus *p16^Xic2^. The arrows indicate the critical PCNA binding amino acids in p21^Cip1^. The rectangle indicates the PIP box sequences and the circle indicates the residue (F123) mutated to disrupt PCNA binding. Left bottom panel: Xic2 GST pull-down assay. GST, GST-Xic2 wildtype (WT), or GST-Xic2 mutants (S98A, F123A, S131A, or T58A) were bound to beads and incubated with LSS. Bound fractions were analyzed by immunoblotting with α-XCyclin E (top) or α-PCNA antibody (bottom). 20% of the input reaction is shown in the left lane (LSS 2ul). Middle panel: Xic2 degradation assay. ^35^S-labeled Xic2 wildtype (WT) or F123A were incubated in LSS with (+) or without (-) 10 ng/ul XSC. The mean percentage of remaining Xic2 from two independent experiments is shown (% Xic2 remaining) where the zero hour time point was normalized to 100%. Right panel: The mean relative ubiquitinated Xic2 WT or F123A from two independent experiments. **C**. PCNA Depletion and Xic2 degradation. Left panel: PCNA immunoblot of HSS not depleted (NOT DEPL), control-depleted (CTRL DEPL), or PCNA-depleted (PCNA DEPL). Middle panel: ^35^S-labeled Xic2 in HSS that was not depleted (NOT DEPL), control-depleted (CTRL DEPL), or PCNA-depleted (PCNA DEPL) with (+) or without (-) ΦX174 (ssDNA, 10 ng/ul). Right panel: The mean percentage of Xic2 remaining from two independent experiments is shown. For all figures, the ubiquitinated Xic2 protein bands are indicated as “XIC2-(UB)n”.

Previous studies identified a requirement for PCNA and PCNA binding for Xic1 turnover and determined the timing of Xic1 turnover was during the DNA polymerase switching step of DNA replication initiation [21]. To examine the timing of Xic2 turnover, we added aphidicolin, an inhibitor of DNA polymerases [21]. Our studies indicated that Xic2 turnover was inhibited by the addition of aphidicolin suggesting Xic2 proteolysis likely requires the activity of DNA polymerase α and the timing of Xic2 degradation occurs following the synthesis of a DNA primer, similar to Xic1 (Figure 3A) [21]. We next identified a consensus PCNA-interacting protein (PIP) box motif (^117^QKLITDFY^124^) within the C-terminus of Xic2 with shared sequence homology to both human p21 and *Xenopus* Xic1 (Figure 3B, left top panel). When we tested the ability of Xic2 to associate with PCNA in the *Xenopus* egg extract, we found that wildtype Xic2 could readily associate with PCNA (Figure 3B, left bottom panel, lane 1). To study a requirement for PCNA binding in Xic2 proteolysis, we mutated a conserved hydrophobic residue of Xic2 within the PIP box (F123) (Figure 3B left panels) and examined this mutant in a degradation assay (Figure 3B, middle and right panels). Past studies have indicated that key conserved hydrophobic residues within the PIP box of p21 and Xic1 are critical for binding to PCNA and consistent with this finding, mutation of Xic2 F123 completely disrupted its ability to bind PCNA in the egg extract (Figure 3B, left bottom panel) [21,27,28]. We found that at the 1.5 and 3 hour time points, the Xic2-F123A point mutant was significantly reduced for ubiquitination compared to wildtype Xic2 (Figure 3C, middle and right panels), suggesting that PCNA binding to Xic2 plays an important role in Xic2 ubiquitination. We also noted a moderate effect on Xic2 turnover (Figure 3B, middle panel). To explore the role of PCNA in Xic2 turnover further, we depleted PCNA from the *Xenopus* extract and studied Xic2 turnover (Figure 3C). The results showed that in the absence of PCNA, Xic2 ubiquitination and turnover were inhibited, again suggesting that PCNA plays an important role in Xic2 proteolysis (Figure 3C, middle and right panels).

### The addition of Cdt2, but not Skp2, promotes the turnover of Xic2

**Figure 4:**
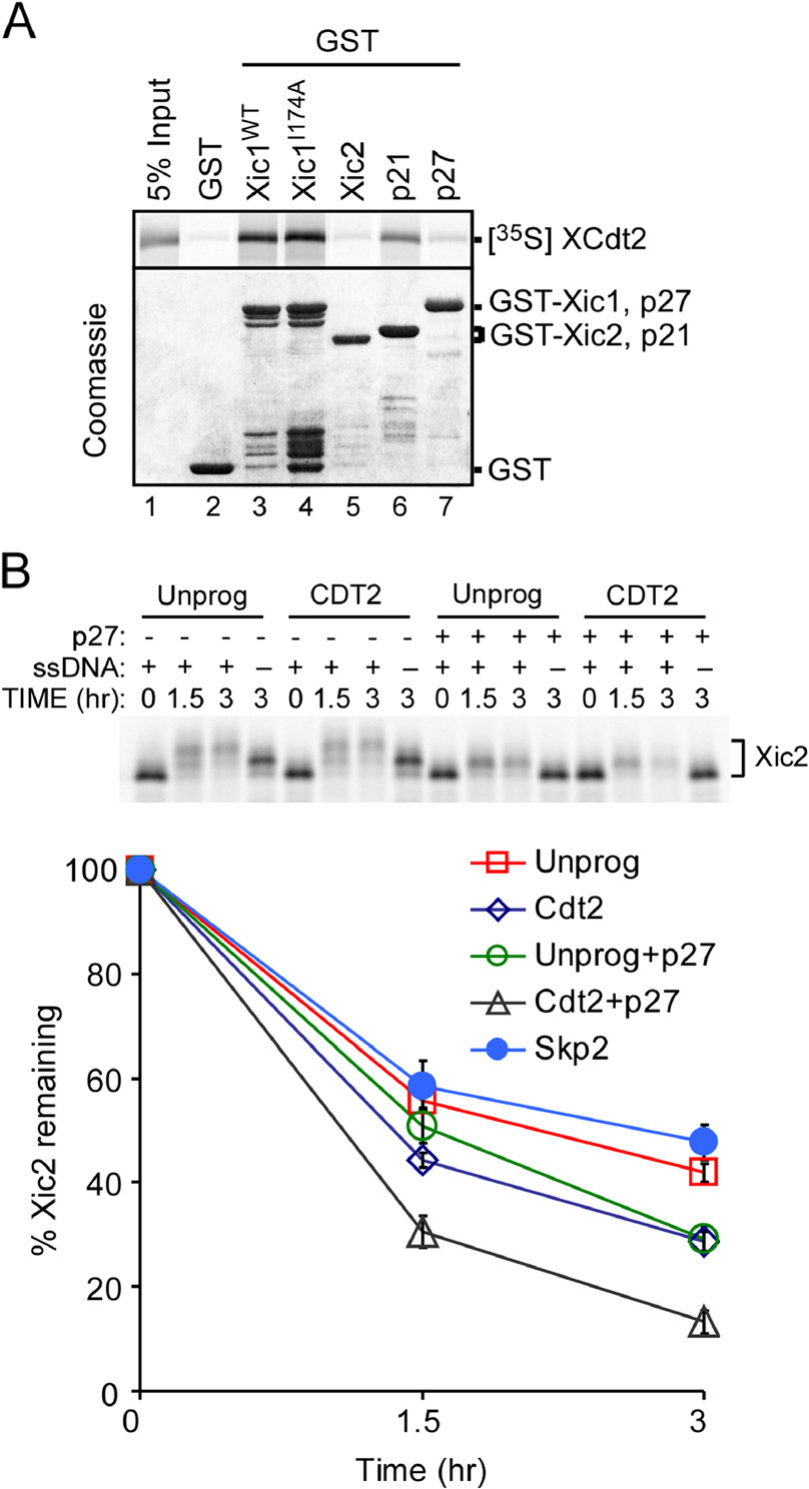
**Cdt2 readily promotes Xic2 turnover in the presence of p27 CDK inhibitor. A**. GST pull-down assay. GST alone or GST fused to wildtype Xic1 (Xic1^WT^), I174A mutant of Xic1 (Xic1^I174A^), Xic2, p21, or p27 (shown in coomassie gel shown in bottom panel) were bound to glutathione sepharose and incubated with in vitro translated ^35^S-methionine labeled *Xenopus *Cdt2 (XCdt2). 5% of the input XCdt2 is shown in lane 1 of the top panel (5% Input). **B**. Xic2 degradation assay. Top panel: ^35^S-methionine labeled Xic2 was incubated in HSS with (+) or without (-) 10 ng/ul ssDNA (ssDNA) in the presence (+) or absence (-) of p27, unprogrammed reticulocyte lysate (Unprog), or unlabeled in vitro translated Cdt2 (CDT2) for 0 to 3 hrs as indicated. Bottom panel: Graphic representation of Xic2 degradation. ^35^S-methionine labeled Xic2 was incubated in HSS with 10 ng/ul ssDNA and the percentage of Xic2 remaining was calculated for each sample where the zero hour time point was normalized to 100%. Reactions were supplemented with unprogrammed reticulocyte lysate (Unprog) (7 experiments), unlabeled in vitro translated Cdt2 (Cdt2) (7 experiments), unprogrammed reticulocyte lysate with p27 (Unprog+p27) (4 experiments), unlabeled in vitro translated Cdt2 with p27 (Cdt2+p27) (4 experiments), or unlabeled in vitro translated *Xenopus *Skp2 (Skp2) (3 experiments). Error bars (Standard error of the mean) are shown. *P* values were calculated by student t-test comparing each sample with the addition of unprogrammed reticulocyte lysate (Unprog). 1.5 hr *p* values: Cdt2 (0.000463), Unprog+p27 (0.270), Cdt2+p27 (0.00184), Skp2 (0.702). 3 hr *p* values: Cdt2 (0.00120), Unprog+p27 (0.0130), Cdt2+p27 (6.56E-05), Skp2 (0.306).

Past studies have indicated that many substrates of the ubiquitin pathway that require PCNA and PCNA binding for their proteolysis are frequently targeted for ubiquitination by the CRL4^Cdt2^ ubiquitin ligase [29,30]. CRL4^Cdt2^ has been shown to ubiquitinate several substrates in a PCNA-dependent manner including Xic1, p21, and Cdt1 [29,30]. Because our studies suggest that the ubiquitination of Xic2 is dependent upon PCNA, we examined whether Xic2 could associate with Cdt2, the substrate binding component of CRL4. Surprisingly, using a GST pull-down assay, our studies indicated that Xic2 did not readily bind to in vitro translated Cdt2 compared to Xic1 and p21 (Figure 4A). To further explore a possible role for CRL4^Cdt2^ in Xic2 turnover, we supplemented the extract with Cdt2 to determine whether this could promote the turnover of Xic2. Our previous studies have shown that for Xic1 turnover, Cdt2 is limiting in the egg extract [20]. Our studies showed that the addition of unlabeled in vitro translated Cdt2 promoted the degradation of Xic2 compared to the addition of unprogrammed reticulocyte lysate, while the addition of in vitro translated Skp2 did not (Figure 4B). Moreover, the concurrent addition of both the CDK inhibitor p27 to prevent Xic2 phosphorylation and Cdt2, significantly promoted the turnover of Xic2, beyond the promotion observed by adding either component individually (Figure 4B). These studies suggest that Cdt2 plays a role in Xic2 ubiquitination and that Xic2 may only associate tightly with Cdt2 in the context of replicating DNA as has been proposed for *Xenopus* Cdt1 [29].

### Xic2 phosphorylation by CDK2 inhibits its proteolysis during interphase

**Figure 5:**
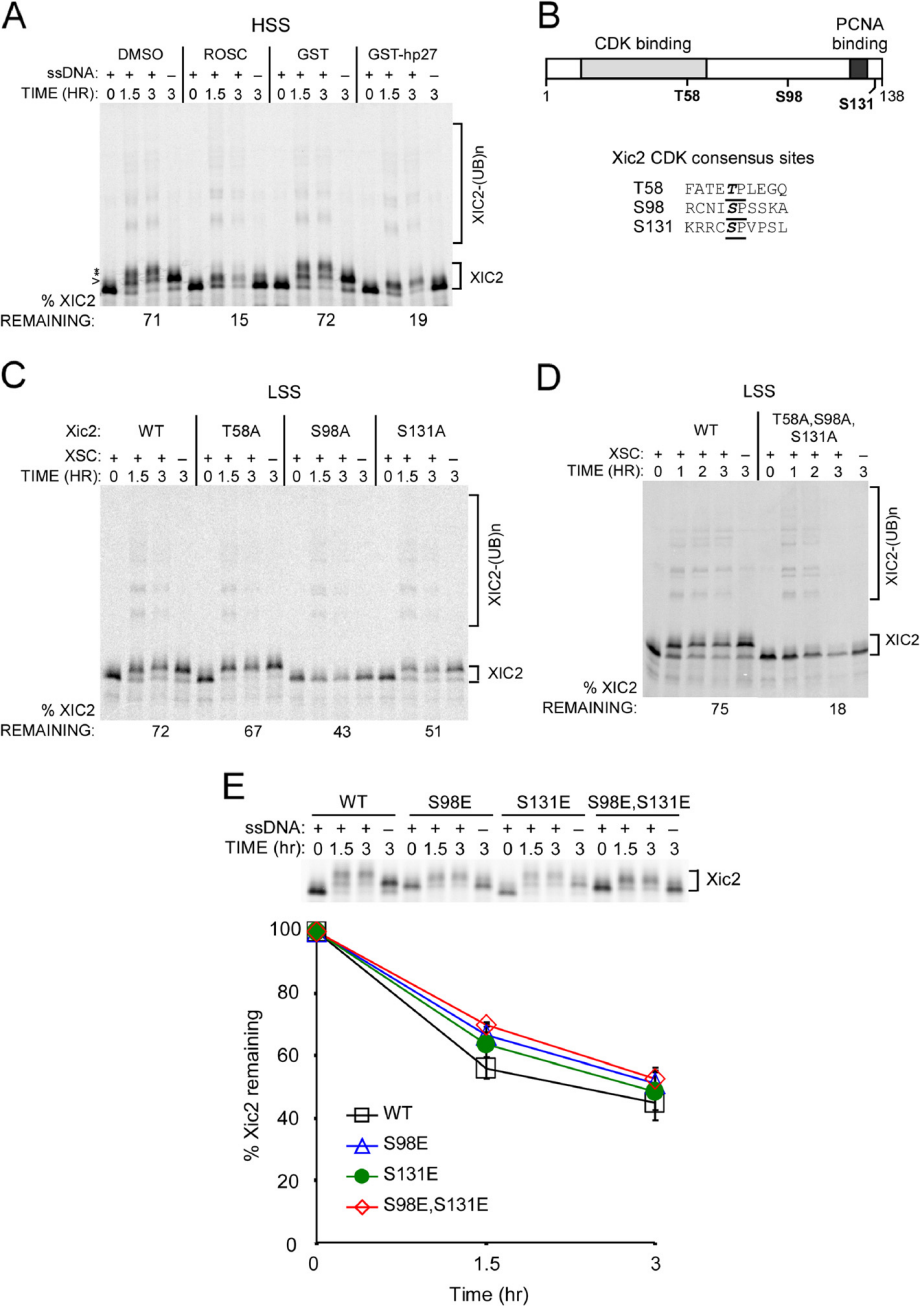
**Phosphorylation of residue S98 by CDK2 stabilizes Xic2. A**. Degradation assay. ^35^S-labeled Xic2 was incubated in HSS with buffer control (DMSO), 1mM roscovitine (ROSC), GST (10uM), or GST-hp27 (10uM) with (+) or without (-) ΦX174 (ssDNA, 10ng/ul). The mean percentage of Xic2 remaining from two independent experiments is shown (% Xic2 remaining) where the zero hour time point was normalized to 100%. **B**. Schematic representation of Xic2 and S/T-P consensus sites. **C**. Xic2 degradation assay. ^35^S-labeled Xic2 wildtype (WT) or CDK phosphorylation mutants (T58A, S98A, or S131A) were incubated in LSS with (+) or without (-) XSC (10ng/ul). The mean percentage of remaining Xic2 from three independent experiments is shown (% Xic2 remaining) where the zero hour time point was normalized to 100%. **D**. Xic2 degradation assay. ^35^S-labeled Xic2 wildtype (WT) or triple-mutant (T58A, S98A, S131A) was incubated in LSS with (+) or without (-) XSC (10ng/ul) for 0 to 3 hrs as indicated. The mean percentage of remaining Xic2 from three independent experiments is shown (% Xic2 remaining) where the zero hour time point was normalized to 100%. **E**. Xic2 degradation assay. Top panel: ^35^S-labeled Xic2 wildtype (WT) or glutamic acid phosphomimetic E mutants (S98E, S131E, or S98E/S131E) were incubated in HSS with (+) or without (-) 10 ng/ul ssDNA (ssDNA) for 0 to 3 hrs as indicated. Bottom panel: The percentage of Xic2 remaining was calculated for each sample where the zero hour time point was normalized to 100%. Error bars (Standard error of the mean) are shown. *P *values were calculated by student t-test comparing each sample with wildtype Xic2 (WT). 1.5 hr *p* values: S98E (0.115), S131E (0.310), and S98E/S131E (0.015). 3 hr *p *values: S98E (0.370), S131E (0.603), and S98E/S131E (0.172). For all figures, the ubiquitinated Xic2 protein bands are indicated as “XIC2-(UB)n”.

Upon examination of Xic2 in the presence of XSC in the *Xenopus* LSS, it is apparent that Xic2 is simultaneously targeted for phosphorylation and proteolysis. However, the proteolysis of Xic2 in the interphase extract is not highly efficient, especially when compared to the proteolysis of Xic1 under the same conditions (Figure 1A). We hypothesized that perhaps the phosphorylation of Xic2 may be negatively regulating the turnover of Xic2. A well-characterized active kinase in the *Xenopus* interphase extract is CDK2-cyclin E [13,31] and to explore whether CDK2 activity may inhibit Xic2 turnover, we examined the effect of CDK2 inhibitors on Xic2 stability. Because CDK2 activity is required for DNA replication initiation in the LSS and Xic2 is degraded during DNA polymerase switching following the CDK2 requirement (Figure 3A), for these studies, it was necessary to use the HSS with single-stranded DNA which supports DNA polymerase switching and Xic2 turnover, but does not require CDK activity [21]. In the presence of either roscovitine or the CDK inhibitor p27, Xic2 turnover in the extract was significantly accelerated compared to the negative controls (DMSO and GST) (Figure 5A). Additionally, in the presence of CDK inhibitors and in absence of DNA, the Xic2 protein bands remained unshifted indicating that the phosphoforms of Xic2 observed in the absence of DNA and CDK inhibitors is due to CDK2 phosphorylation (Figure 5A).

To directly test the role of CDK2 phosphorylation on Xic2 stability, we mutated the three possible CDK consensus sites within Xic2 individually and in combination to alanine to prevent phosphorylation (Figure 5B, C, D). Mutation of residue Thr-58 (T58) had little effect on Xic2 turnover, while mutation of residues Ser-98 (S98) or Ser-131 (S131) both resulted in more efficient proteolysis of Xic2 (Figure 5C). Additionally, mutation of S98 abolished phosphoform 1 of Xic2 suggesting that phosphorylation of Xic2 at residue 98 is responsible for the shifted phosphoform 1 of Xic2 in the interphase extract (Figure 5C). Simultaneous triple mutation of Xic2 at residues T58, S98, and S131 resulted in a Xic2 mutant that did not exhibit a shift and was efficiently degraded in the interphase extract compared to wildtype Xic2 (18% Xic2 remaining for the T58A, S98A, S131A mutant compared to 75% remaining for WT Xic2) (Figure 5D). We further generated glutamic acid mutations of Xic2 at residues S98 and S131 to mimic constitutive phosphorylation and tested these Xic2 mutants for turnover. Our studies indicated that the Xic2-S98E, S131E double mutant and the S98E and S131E single point mutants were all degraded similarly or less efficiently than the wildtype Xic2 that is phosphorylated in the extract (Xic2 WT and the E mutants are degraded to approximately 50% of Xic2 remaining, Figure 5E, versus 18% for the Xic2 triple alanine mutant that is not phosphorylated, Figure 5D). This result is consistent with our hypothesis that Xic2 phosphorylation in the extract inhibits its turnover. Taken together, these studies suggest that CDK2-dependent phosphorylation of Xic2 at residues S98 and S131 negatively regulates its ubiquitination and degradation during interphase.

### Xic2 is hyperphosphorylated at residues S78/S81 in a manner dependent upon single-stranded DNA and a caffeine-sensitive kinase

**Figure 6:**
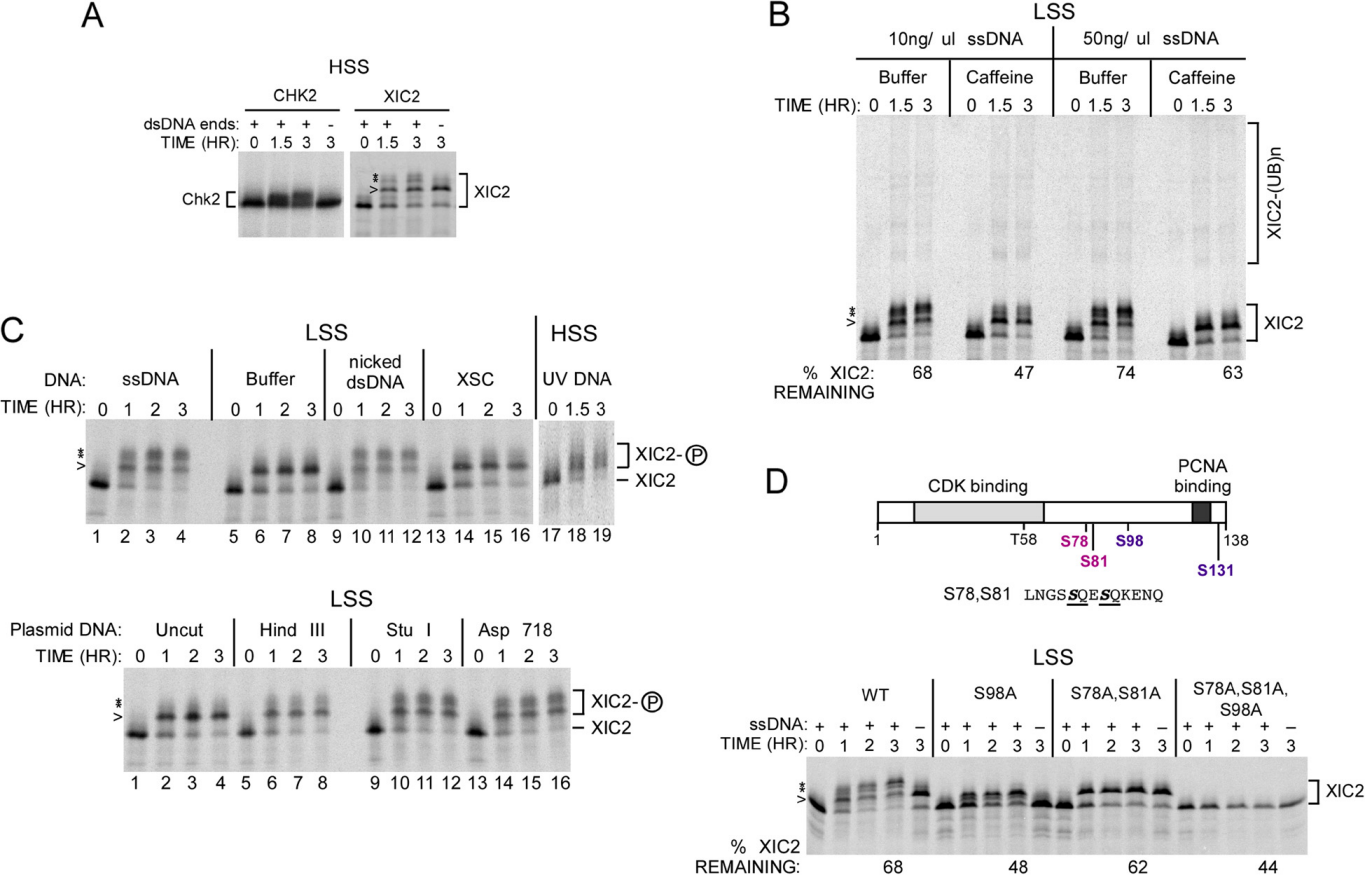
**Single-stranded DNA-dependent Xic2 phosphorylation at residues S78/S81 is sensitive to caffeine treatment. A**. Phosphorylation shift assay. ^35^S-methionine labeled Chk2 and Xic2 were incubated in HSS with (+) or without (-) annealed oligonucleotides DNA (dsDNA ends, 100 ng/ul) for 0 to 3 hrs as indicated. **B**. Xic2 degradation assay. ^35^S-methionine labeled Xic2 was incubated in LSS with 10 ng/ul or 50 ng/ul ΦX174 single-stranded DNA (ssDNA) in the presence of XB- (buffer) or 10 mM caffeine for 0 to 3 hrs as indicated. The mean percentage of remaining Xic2 from two independent experiments is shown (% Xic2 remaining) where the zero hour time point was normalized to 100%. **C**. Phosphorylation shift assay. ^35^S-methionine labeled Xic2 was incubated in LSS or HSS as indicated with ΦX174 (ssDNA), XB- (Buffer), nicked pCS2+ plasmid DNA (nicked dsDNA), XSC, UV-irradiated plasmid DNA (UV DNA), uncut plasmid DNA (Uncut), HindIII linearized plasmid DNA (HindIII), StuI linearized plasmid DNA (StuI), or Asp718 linearized plasmid DNA (Asp718) at 10 ng/ul final DNA concentrations for 0 to 3 hrs as indicated. **D**. Xic2 degradation assay. Top panel: Schematic representation of Xic2 with potential S/T-P and S/T/-Q phosphorylation sites and the proximal sequences surrounding the S/T-Q sites of Xic2. Bottom panel: ^35^S-methionine labeled Xic2 wildtype (WT) or mutants (S98A, S78A/S81A, or S78A/S81A/S98A) were incubated in LSS with (+) or without (-) 10 ng/ul ΦX174 single-stranded DNA (ssDNA) for 0 to 3 hrs as indicated. The mean percentage of remaining Xic2 from two independent experiments is shown (% Xic2 remaining) where the zero hour time point was normalized to 100%. In all figures, the caret (<) and asterisks (*) indicate the slower migrating phosphoforms of Xic2 and phosphorylated Xic2 is also indicated as XIC2-P.

In the interphase egg extract, in addition to the DNA-independent phosphorylation of Xic2 by CDK2-cyclin (Figures 2 and 5, phosphoform 1), we have also observed an additional DNA-dependent shift in the presence of single-stranded DNA (Figure 2, B and C, phosphoforms 2). The DNA-dependent phosphorylation of Xic2 causes a doublet of Xic2 to appear in the presence of single-stranded DNA with a slower migration than phosphoform 1 resulting from CDK2 phosphorylation of residue S98 (Figure 2B, C, D and Figure 5). Previous studies have indicated that single-stranded DNA in the interphase egg extract is replicated to form double-stranded DNA ends which trigger activation of the checkpoint kinase, Ataxia Telangiectasia-related protein (ATR), and induce the phosphorylation of Chk2 [32,33]. Consistent with these previous findings, we observed that the timing of the ssDNA-dependent mobility shift of Xic2 coincided with the appearance of a Chk2 shift in the presence of double-stranded DNA (dsDNA) ends (Figure 6A). Moreover, the Xic2 shifts observed in the presence of ssDNA or replicated dsDNA ends (phosphoforms 2) was prevented by the addition of caffeine, a known inhibitor of Class IV Phosphatidylinositol 3-kinases (PI 3-kinases or PI3Ks) such as ATR [32,33], suggesting that an ATM/ATR-like kinase may be responsible for phosphorylating Xic2 in the presence of ssDNA (Figure 6B). We also noted a modest increase in Xic2 turnover in the presence of caffeine perhaps indicating that the phosphorylation of Xic2 in the presence of ssDNA may be partially stabilizing (Figure 6B). To further characterize the modification of Xic2 in the presence of ssDNA, we examined Xic2 in the presence of a variety of DNA templates in the *Xenopus* interphase egg extract. Our studies showed that Xic2 did not exhibit the “phosphoforms 2” modification in the presence of buffer, XSC, or uncut plasmid DNA (Figure 6C). In contrast, Xic2 phosphoforms 2 were observed to varying degrees in the presence of ssDNA, nicked dsDNA, UV-damaged DNA, and linearized plasmid DNA (Figure 6C). These studies suggest that damaged DNA templates or DNA templates which mimic damaged DNA activate a caffeine-sensitive kinase that phosphorylates Xic2. Notably, in the presence of aphidicolin, these caffeine-sensitive modifications of Xic2 are inhibited (Figure 3A).

An examination of the potential sites targeted for phosphorylation of Xic2 in the presence of ssDNA revealed two residues at S78 and S81 that matched the S/T-Q consensus site for ATM-ATR-like kinases (Figure 6D, top). Mutation of these two residues to alanine (S78A, S81A) completely eliminated the Xic2 phosphoforms 2 in the presence of ssDNA compared to wildtype Xic2 (Figure 6D, bottom, samples for “S78A, S81A” and “S78A, S81A, S98A”). Phosphorylation of Xic2 by the caffeine-sensitive kinase in the presence of ssDNA does not appear to be dependent upon prior or concurrent phosphorylation of Xic2 by CDK2-cyclin as shown by the shift of a Xic2-S98A mutant (Figure 6D) and by the ability of Xic2 to be phosphorylated in an ssDNA-dependent manner in the presence of roscovitine (data not shown). This suggests that the interphase phosphorylation of Xic2 by CDK2 and the caffeine-sensitive kinase function along independent pathways.

## Discussion

**Figure 7:**
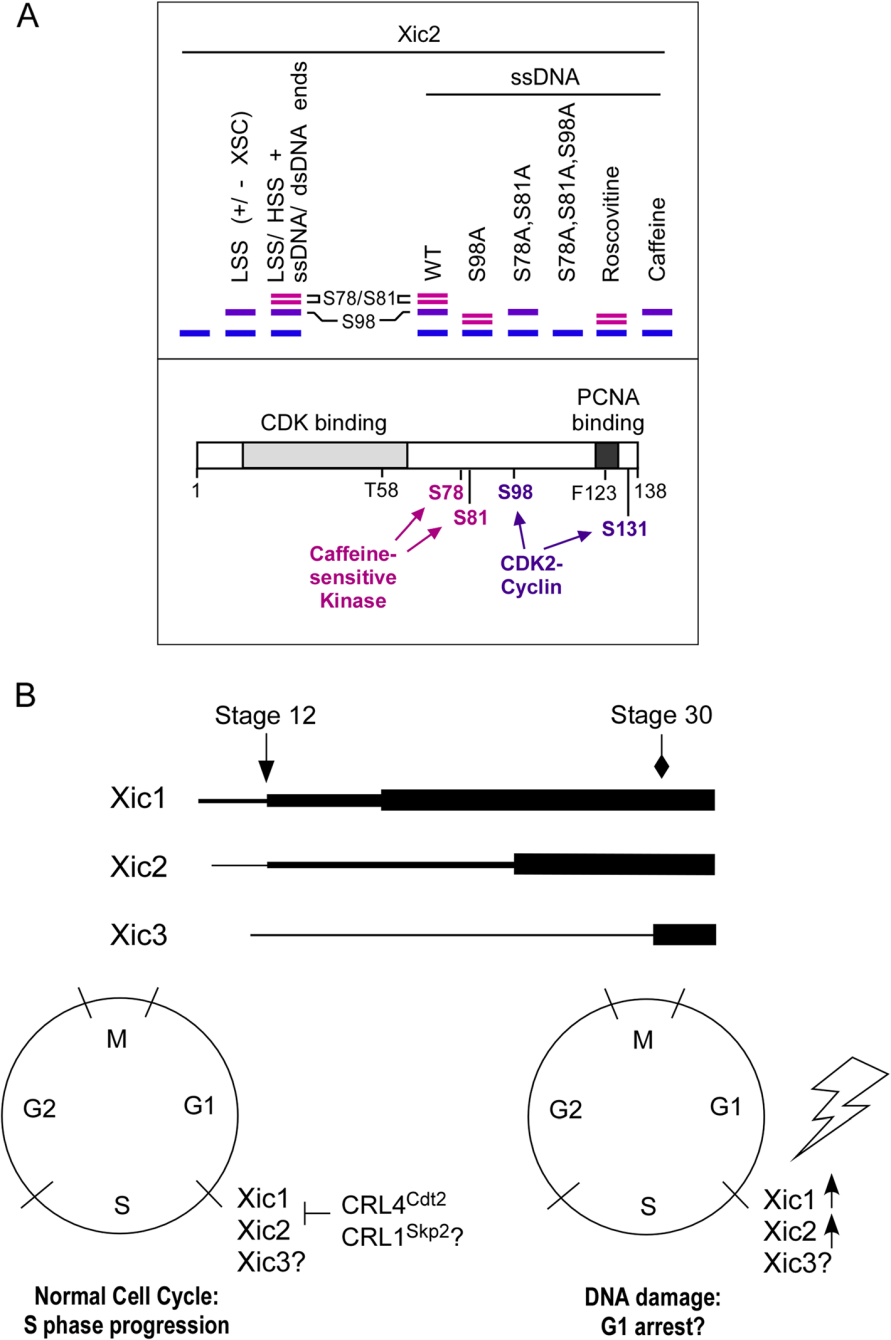
**Idealized representation of Xic2 phosphoforms, developmental expression, and cell cycle regulation. A**. Schematic representation of ^35^S-methionine labeled Xic2 wildtype and mutant protein bands or Xic2 under specific conditions (untreated, in extract, with DNA, or with kinase inhibitors) (top panel) and schematic representation of Xic2 phosphorylation sites targeted by CDK2-cyclin and a caffeine-sensitive kinase (bottom panel). Unphosphorylated Xic2 is marked by the blue line, S98 phosphorylated Xic2 is marked by the purple line, and S78/S81 phosphorylated Xic2 bands are marked by the pink lines. **B**. Top Panel: Schematic representation of Xic1, 2, 3 RNA/protein expression during *Xenopus *development where the thicker lines represent higher expression, the arrow indicates the timing of gastrulation (stage 12) and the diamond indicates the tailbud stage (30). Bottom panel: Xic1, 2, and 3 are predicted to function at the G1 to S phase transition during development and in a normal somatic cell cycle as shown in the drawing (left). It is predicted that Xic1 and Xic2 are targeted by CRL4^Cdt2 ^and CRL1^Skp2 ^ubiquitin ligases. During a response to DNA damage caused by IR (right), both Xic1 and Xic2 are predicted to be upregulated to halt the cell cycle in G1 during a checkpoint to allow DNA repair to occur.

In the initial description of Xic2, the expression of Xic2 RNA was described to be initially low during stages 10–11, increasing at stage 18, with the highest expression at stage 25 with staining appearing predominantly in the developing somite, tail bud, lens, and cement gland suggesting a role in developmental patterning (Figure 7B, top) [11]. This is in contrast to the expression of Xic1 which is observed earlier during development and increases significantly following gastrulation (Figure 7B, top) or the expression of Xic3 that is very low until after stage 28 with a peak at stage 38 indicating that Xic3 functions during late embryonic patterning (Figure 7B, top) [11,12,14,16,24]. Xic3 was found to be stable in the interphase egg extract suggesting that the machinery or regulators that control Xic3 stability may not be appreciably expressed or activated in the *Xenopus* egg.

Our studies suggest that like mammalian p21, Xic2 protein levels appear to be induced upon treatment with IR [26]. This result suggests that in addition to a role in development, Xic2 may also play a role in a DNA damage checkpoint with Xic2 levels increasing during DNA damage, similar to Xic1, to presumably halt entry into S phase to allow DNA repair to occur (Figure 7B, bottom right) [34]. Consistent with this hypothesis, Xic2 appears to be phosphorylated by a caffeine-sensitive kinase in the presence of double-stranded DNA ends, a signal shown to activate a DNA damage checkpoint in the interphase egg extract [32,33] (Figure 7A). Under the same conditions, XCds1 (*Xenopus* Chk2) has been shown to be phosphorylated and this phosphorylation is inhibited by a block to DNA replication and by caffeine suggesting Xic2 may be modulated by the same checkpoint pathway as XCds1 [32]. How the phosphorylation of Xic2 at residues S78 and S81 may influence its CDK2 inhibitory activity or binding to CDK2 and PCNA are currently under further investigation.

The fact that Xic2 is readily ubiquitinated and degraded in the interphase egg extract when CDK2 phosphorylation is inhibited suggests that the ubiquitination machinery that targets Xic2 for degradation is present in the egg. Our past studies of the *Xenopus* interphase egg extract suggest that the CRL4^Cdt2^ ubiquitin ligase is active in the extract while the SCF^Skp2^ ubiquitin ligase is not due to very low expression of Skp2 in the egg [20,24]. Our results demonstrating the dependence of Xic2 proteolysis on PCNA and DNA, the timing of Xic2 ubiquitination and degradation, and the ability of Cdt2 to promote Xic2 turnover are all consistent with CRL4^Cdt2^ being the ubiquitin ligase of Xic2 in the egg extract. It is then somewhat puzzling that Xic2 does not readily bind to Cdt2 under conditions that efficiently support Xic1 and Cdt2 binding. Similar to p21 and Xic1, Xic2 bears the “specialized PIP box” described by Havens and Walters [29,35] which contains a PIP box that binds PCNA with high affinity (contains the TD motif) followed by a basic residue at the +4 position relative to the PIP box. Our findings suggest that Xic2 may more closely resemble the replication protein and CRL4^Cdt2^ substrate, Cdt1, in requiring the presence of DNA to appreciably bind to Cdt2. However, unlike Cdt1, Xic2 readily binds PCNA in the absence of DNA. It is also possible that Xic2 is only indirectly targeted by Cdt2 or that Xic2 is additionally targeted by an alternative E3 in the egg extract, although our studies indicated that the addition of Skp2 did not promote Xic2 turnover. It is interesting to note that sequence analyses revealed a KEN box present in both Xic2 (^83^KENQCQD^89^) and Xic1 (^138^KENAEKI^144^) suggesting that Xic2 and Xic1 may be targeted for ubiquitination by the ubiquitin ligase, APC^Cdh1^. However, Cdh1 expression in the *Xenopus* egg is very low and it is not appreciably expressed until after gastrulation [36].

Using the *Xenopus* model extract system, we have evidence to support that Xic2 is phosphorylated in a CDK2 dependent manner, most likely by CDK2-cyclin E, the predominant CDK activity in the interphase egg extract [31] (Figure 7A). The phosphorylation of Xic2 at residues S98 and S131 appear to equally inhibit the ubiquitination and degradation of Xic2 in the extract. The inhibition of Xic2 turnover by CDK2 phosphorylation may be due to a change in the cell localization of Xic2 upon phosphorylation although this is unlikely to be the predominant contributing factor since our degradation studies using the HSS extract which does not contain nuclear membrane precursors and does not support nuclei formation [37] still showed that CDK2 phosphorylated Xic2 was inhibited for degradation. Alternatively, Xic2 phosphorylation by CDK2 may influence the binding of Xic2 to PCNA, DNA, or the ubiquitination machinery. The finding that CDK2 phosphorylation of Xic2 inhibits its PCNA-dependent turnover suggests that Xic2 phosphorylation may be an important regulator of Xic2 stability and function.

## Conclusions

In this study, we provide the first biochemical examination of the regulation of the *Xenopus* CDK inhibitors, p16^Xic2^ and p17^Xic3^, using the egg extract model system. Our studies indicate that Xic2 is targeted for DNA- and PCNA-dependent ubiquitination and degradation in the interphase egg extract and that this turnover of Xic2 is promoted by Cdt2 and inhibited by CDK2-dependent phosphorylation of Xic2 at residues Ser-98 and Ser-131. Additionally, it appears that during conditions mimicking a DNA damage checkpoint, Xic2 is targeted for phosphorylation by a caffeine-sensitive kinase at residues Ser-78 and Ser-81, although the consequence of this phosphorylation is still unclear. Xic3 appears to be stable in the interphase egg extract in the presence or absence of DNA.

In their initial discovery of Xic2, Daniels et al. [11] described Xic2 as an ortholog of mammalian p21 which is known to be transcriptionally induced by p53 upon DNA damage. p21 has also been shown to be a substrate of both CRL4^Cdt2^ and SCF^Skp2^[38-40]. The RNA expression pattern of Xic2 in somites, the tail bud, the lens, and the cement gland suggest that Xic2 protein is expressed during late embryonic development [11], but how Xic2 protein may be regulated by proteolysis during development remains unknown. It will be important to study how Xic2 may be regulated by PCNA, CRL4^Cdt2^, or CDK2 during developmental patterning. It will also be necessary to study Xic1, Xic2, and Xic3 and their regulators in the context of the developing embryo and the somatic cell to fully understand how these three *Xenopus* CDK inhibitors mediate the events of early development and cell cycle control in the frog.

## Methods

### Preparation of *Xenopus* extracts and demembranated sperm chromatin

*Xenopus* interphase extracts [low speed supernatant (LSS) and high speed supernatant (HSS)] [23], stable Δ90 mitotic extract (LSS supplemented with Δ90 non-degradable cyclin B), and demembranated *Xenopus* sperm chromatin (XSC) [19,41,42] were prepared as previously described. All studies involving animals were conducted according to the rules established by the Universities of Federation for Animal Welfare, the World Society for the Protection of Animals Working Party, and the American Veterinary Medical Association. This work was approved by the Institutional Animal Care and Use Committee of the University of Texas Health Science Center at San Antonio which is accredited by the Association for Assessment and Accreditation of Laboratory Animal Care under protocols 99045I-04-06-A and 11073x.

### Cell culture

*Xenopus* Tissue Culture (XTC) cells were propagated at room temperature in L-15 medium with L-glutamine (Sigma-Aldrich) supplemented with 10% fetal bovine serum (Gibco-BRL) and 50 ug/ml of penicillin-streptomycin (Gibco-BRL). Cells were irradiated with 10 Gy using a ^137^Cesium source Mark I Model 68A irradiator and harvested 4 and 8 hours later in RIPA buffer (10 mM Tris-Cl, pH 8.0, 1mM EDTA, 0.15 M NaCl, 1% Np-40 and 1% Sodium Deoxycholate) containing protease inhibitors (Sigma, P8340).

### Generation of Xic2 mutants and other constructs

All point mutants of Xic2 in pCS2+ were generated by using pCS2+-Xic2 as the template and the QuickChange™ Site-Directed Mutagenesis Kit (Stratagene) followed by DNA sequencing to confirm the mutagenesis. Mutagenesis primer sequences are available upon request. pGEX4T-Xic2 was generated by subcloning a PCR fragment of Xic2 using pCS2+-Xic2 as the template into the BamHI and SalI restriction sites of pGEX4T-1.

### Production of Xic2 antibody and other antibodies

GST-Xic2 expressed in BL21Star (DE3) was purified using Glutathione-Sepharose 4B beads (Amersham Biosciences) according to the manufacturer’s instructions. Rabbit polyclonal antibody against GST-Xic2 was generated by the University of Texas Health Science Center at San Antonio SACI antibody core facility. Anti-PCNA mouse monoclonal antibody was purchased from Santa Cruz (P-10) and anti-GST-XCyclin E antibody was a gift from Peter K. Jackson and Marc W. Kirschner.

### In vitro transcription and translation

*In vitro* transcription and translation reactions were performed using the SP6 TNT coupled reticulocyte lysate system (Promega) and ^35^S-methionine from New England Nuclear.

### Dephosphorylation and inhibition assays

Dephosphorylation of protein samples was performed using lambda phosphatase (λ-PPase) (New England Biolab, P0753S). Reactions were incubated at 30°C for 30 min in the presence of 80 or 200 units of λ-PPase, terminated by the addition of protein loading dye, and analyzed by SDS-PAGE and phosphorimager. Roscovitine (A. G. Scientific Inc, R-1016) was used as previously described to inhibit CDK2 activity [21]. Caffeine (Sigma) was used at a final concentration of 10 mM from fresh stocks of 75 mM in XB- (100 mM KCl, 1 mM MgCl_2_, 0.1 mM CaCl_2_, 10 mM HEPES pH 7.7).

### GST pull down assays, immunoblotting, and immunoprecipiation

GST pull down assays using egg extract and immunoblotting were performed as previously described except the binding reactions were conducted at 4°C for 2 hours [21,22]. Rabbit serum against Xic2 was used to immunoprecipitate and immunoblot Xic2 in extracts. Anti-cyclin E antibody was used to immunoprecipiate cyclin E from extracts while anti-PCNA antibody was used for immunoblots. Normal rabbit serum was used as a control for immunoprecipitations.

### Degradation assay, nuclei spin down assay, and phosphorylation shift assay

Degradation assays were performed as previously described [21] with the following modifications. Proteins labeled with ^35^S-methionine were added to extracts at a final dilution of 1:15 in the presence or absence of 10 ng/ul demembranated XSC or ΦX174 single-stranded DNA (New England Biolab, N3023S). The reactions were analyzed by PhosphorImager and quantitation was performed using ImageQuant™ software (Molecular Dynamics). The percentage of protein remaining for each sample was determined by normalizing the amount of protein at the 0 hr time point to 100%. Nuclei spin down assays were performed as previously described [23,24] with the following minor modifications. LSS was incubated with ubiquitin (3 ug/ul), ^35^S-methionine labeled Xic2 at a final dilution of 1:15, and XSC (10 ng/ul) for 90 min at 23°C. When indicated, methyl ubiquitin was added to a final concentration of 3 ug/ul. Ubiquitin (Sigma) was methylated as previously described [25]. In vitro translated Chk2 (XCds1-pCS2+) or Xic2 was added to HSS and incubated for 0 to 3 hours in the presence or absence of double-stranded DNA ends that were generated from annealed oligonucleotides [43].

## Abbreviations

CDK: Cyclin-dependent kinase; LSS: Low speed supernatant; HSS: High speed supernatant; XSC: *Xenopus* sperm chromatin; PCR: Polymerase chain reaction; DMSO: Dimethyl-sulfoxide; GST: Glutathione S-transferase; ROSC: Roscovitine; Xic2: p16^Xic2^; Xic1: p27^Xic1^; PCNA: Proliferating cell nuclear antigen; SCF: Skp1-Cullin-F-box; CRL: Cullin-RING ligase; Kix1: p28^Kix1^; a.a.: Amino acid; SDS: Sodium dodecyl sulfate; PAGE: Polyacrylamide gel electrophoresis; CIP: Calf intestinal phosphatase; RRL: Rabbit reticulocyte lysate; WT: Wildtype; IVT: *In vitro* translated.

## Competing interests

The authors declare that they have no competing interests.

## Authors’ contributions

HRL initiated the Xic2 and Xic3 studies and contributed to Figures 1 and 2; XNZ performed the Xic2 proteolysis and phosphorylation studies, generated the majority of the data, and participated in drafting the manuscript; DHK performed statistical analyses, performed the Cdt2 studies of Figure 4, and contributed to Figures 3, 5, and 6; VNB generated UV-damaged DNA and contributed to Figure 6; HBR generated the Xic2 glutamic acid mutants and contributed to Figure 5; PRM participated in study design and data analyses; PRY directed the study, participated in its design and coordination, and drafted the manuscript. All authors read and approved the final manuscript.
